# Milk Yield, Milk Composition, and the Nutritive Value of Feed Accessed Varies with Milking Order for Pasture-Based Dairy Cattle

**DOI:** 10.3390/ani9020060

**Published:** 2019-02-14

**Authors:** Kamila Dias, Sergio Garcia, Mohammed (Rafiq) Islam, Cameron Clark

**Affiliations:** 1 Animal Science Department, Santa Catarina State University (UDESC), Lages SC 88520-000, Brazil; kamila.dias@gmail.com; 2 Dairy Science Group, School of Life and Environmental Sciences University of Sydney, Camden, NSW 2570, Australia; sergio.garcia@sydney.edu.au (S.G.); md.islam@sydney.edu.au (M.I.)

**Keywords:** milking order, dairy, pasture, precision farming

## Abstract

**Simple Summary:**

In pasture-based dairy systems, dairy cattle that voluntarily walk back to pasture immediately after milking access greater feed nutritive value than those cattle milked last. Dairy cattle that were milked first in our work produced more milk and tended to have a greater yield of milk solids than cattle milked last. This work highlights the opportunity to improve nutrient use efficiency on dairy farms through strategic pasture allowance and supplementation.

**Abstract:**

(1) Background: Pasture varies in its chemical composition from the top of the sward to the base and cattle prefer to eat the leaf fraction. In pasture-based dairy systems, cattle predominantly walk back to pasture voluntarily after each milking, with the first cattle arriving to pasture hours before the last. Here we study the impact of pasture composition according to milking order on milk yield and milk composition for dairy cattle offered grazed ryegrass pasture. (2) Methods: In the first experiment, individual cow milk yield data were recorded on six farms over 8 months. The herd was divided into groups of 50 cows for analysis according to milking order. In the second experiment, the impact of milking order on milk composition and pasture composition accessed was determined in addition to milk yield on three farms. (3) Results: After accounting for age and stage of lactation effects, cattle milked first in experiment 1 produced, on average, 4.5 L/cow/day (+18%; range 14 to 29%) more than cattle milked last. In experiment 2, dairy cattle milked first (first 50 cows) in farm 1 had greater milk, protein, and solids non-fat (SNF) yield; and less lactose content than those milked last (last 50 cows). In farm 2, dairy cattle milked first had greater milk yield, SNF yield, lactose yield, and fat yield; but less protein and SNF content than cattle milked last. In farm 3, cattle milked first produced milk with greater fat and protein content than cattle milked last. In line with these differences in milk yield and composition, the composition of pasture across vertical strata differed, particularly for crude protein (CP) and acid detergent fiber (ADF) content. Conclusion: This work highlights the opportunity to increase herd nutrient use efficiency for improved milk production through strategic pasture allowance and supplementation strategies.

## 1. Introduction

Grazed pasture is a low-cost feed when managed well, and as such, is widely used in Australia as well as in many parts of the world [1]. The depletion of this pasture sward canopy by cattle typically occurs in successive layers [2,3], which varies in chemical composition with the top of the fraction typically containing more crude protein (CP) and less neutral detergent fiber (NDF) than lower fractions [4,5]. This pasture composition is related to the proportion of leaves, which can decrease as sward surface height of pasture decreases [6]. 

In pasture-based dairy systems, farmers typically allow dairy cattle to voluntarily walk back to pasture after the cattle are milked. For these systems, the dairy cattle access pasture at varying states of differing nutritive value [5], and as milking order is consistent [5,7,8], dairy cattle will access a similar state of pasture after each milking. Further, the state of pasture accessed by dairy cattle impacts milk production levels with dairy cattle offered fresh, non-depleted pasture producing approximately 10% greater milk yield than those cattle offered the same temperate or tropical pasture at a depleted state [9]. 

In retrospective data analyses, some studies have shown a strong association between milk yield and milking order [8,10,11], and others a weak association between milking order and milk yield [12,13]. Research is required to elucidate the differences in findings between previous work, with a key being to investigate all variables in the same study, namely milking order, pasture quality and pasture state accessed, milk production, and milk composition. In addition, pasture-based dairy farms are intensifying, with herd sizes increasing, and as such the differences in pasture nutritive value accessed by cattle milked first and last may be increasing. The overall objective of our work was to determine the impact of milking order and diet composition (pasture quality, pasture mass, and levels of concentrate) on milk yield and milk composition for large dairy herds as a key step towards the implementation of precision systems to optimize milk production from pasture. Two experiments were conducted to complete this objective. Experiment 1 determined the impact of milking order on milk yield for large pasture-based herds, and experiment 2 determined the impact of milking order and diet composition on milk yield and milk composition for large dairy herds.

## 2. Materials and Methods

### 2.1. Experiment 1

Daily milk yield and grain-based concentrate data from January 2015 to August 2015 were determined for six dairy farms from Tasmania, Australia (see Table 1). Cattle on these farms were of predominantly Holstein–Friesian breed and milked at 0500h and 1500h daily. These farms were selected based on farm size, system, and milking equipment with each equipped with a rotary milking parlor (50 to 60 bales) and using the ALPRO (DeLaval) software system. The pasture offered to dairy cattle comprised predominantly perennial ryegrass for all farms and were between 1 and 1.5 km (maximum) from the milking parlor.

### 2.2. Experiment 2

In October 2015, the first and last 50 cows milked (as per milking order) on three of these farms (see Table 2) were selected and had milk samples collected for three consecutive days. 

Both milk and plant samples were collected across three consecutive days for each farm. This collection was staggered so that only pasture samples were taken on day 1, pasture and milk samples on days 2 and 3, and only milk samples on day 4. 

Fifty mL of milk per cow per day was stored at 4 °C with preservative (bronopol) until analyzed for fat, protein, lactose, and solids non-fat (SNF) by infrared analysis (Fourier Transform Spectrometer (FTS), Bentley Instruments FTS/FCM 500 Combi) and somatic cells by fluorescence cytometry analysis (Flow Cytometer (FCM)) at TasHerd laboratory (Tasmania; Herd Test Centre). Standards were supplied by Global Proficiency in Melbourne (for fat, protein, lactose, and SNF) and by the Victorian Department of Environment and Primary Industries laboratory at Ellinbank in Victoria (for somatic cells).

Pasture height (extended tiller) was evaluated using a ruler in experiment 2 for morning pasture allocations pre-grazing, post-grazing, and every 15 min between the first and last cow arriving to the paddock. Herbage mass was measured daily during the experiment at nine random sites in the pre- and post-grazing areas. To determine herbage mass offered to cattle in each paddock, all plant material within nine randomly allocated quadrats (30 × 30 cm) was harvested to ground level using a cordless grass shearer (RYOBI-RGS182Li15), the fresh weight was recorded, and each quadrat was frozen separately before drying at 60 °C for 48 hours. The dry matter yield of each quadrat was used to estimate the herbage mass on pre- and post-grazing areas (average of all quadrats). According to paddock size and number of cows per paddock, the pre-grazing herbage mass was used to calculate the herbage allowance (HA, kgDM/ha to ground level). Apparent dry matter intake (DMI; kg/cow per day) was calculated from the difference between pre- and post-grazing pasture mass and the number of cows grazing a given area.

The vertical structure of the pre-grazing samples was maintained, labelled, and taken for chemical composition analysis. All pre-grazing samples were defrosted, pooled based on the day of collection, cut into 2 cm fractions, and labelled according to stratum level (0 to 2 cm from ground level: stratum 1; 2 to 4 cm: stratum 2, etc.). A representative sample of each quadrat was used to determine the botanical composition and dry matter content. For botanical composition, the pasture was separated into ryegrass, clover, and other species. The stratified pasture samples and grain were dried at 60 °C for 48 hours, ground to ~1mm, and then analyzed for crude protein (CP, FP628 Food/Protein Analyzer, LECO, Michigan, USA) and acid detergent fiber (ADF, ANKOM200 Fiber Analyzer, ANKOM, New York, USA). Organic matter digestibility (OMD) and metabolizable energy (ME) were estimated according to Ketelaars et al. [14].

### 2.3. Statistical Analysis

For experiment 1, cattle were classified in milking order groups of 50 animals for the morning and afternoon milking periods. Daily milking order was the average of the morning and evening milking order. Animals without recordings were considered missing values. Period (week of the year), parity, milking order, and concentrate intake (groups: 0–3.0 kg, 3.1–6.0 kg) were factors in the model, ‘cow’ was included as a random effect, and days in milk (DIM) as a covariate, using the statistical program SAS, MIXED procedure. Individual cows were offered grain-based concentrate based on feed tables according to level of milk production. Individual cows were considered as an experimental unit and period as a repeated measurement. To determine the effects of milking order (MO) (11 to 24 groups accounting for variation in cow number through time), period (P) (31 groups including data from the start of January to the last week in August), parity (PA), DIM (D), and concentrate intake (C) (2 groups) on milk yield (MY) (Model 1) a mixed linear model was fitted to the data as follows:

MYijklm = μ + MOi + Pj + PAk + Cm + b1(D)ijklm + εijklm(1)

where MY is the dependent variable, μ is the overall mean, b1(D)ijklm is days in milk as a covariate and εijklm is the random experimental error.

For experiment 2, milk yield and components for the first and last 50 cattle and pasture results were analyzed using the statistical program SAS, MIXED procedure. ‘Cow’ was included as a random effect; individual cows were considered as an experimental unit, and day as a repeated measurement. Days in milk, parity, and grain intake were excluded in the model, as they were not significant in the preliminary model (*p* > 0.05). To determine the association between milking order (MO) (first and last 50 cows) and milk yield (MY), milk component (M) or pasture composition (P), a mixed linear model was fitted to the data as follows:

MYi or Mi or Pi = μ + MOi + εi(2)

where MY, M or P are the dependent variables, μ is the overall mean, and εi is the random experimental error. 

## 3. Results

### 3.1. Experiment 1

There was a strong association between morning and afternoon milking order (y = 0.51 + 0.92x; R^2^ = 0.87, *p* < 0.001). Milking order was negatively associated (*p* < 0.01) with milk yield in all farms. The predicted mean milk yield difference between the first 50 and last 50 cows for all farms was 4.5 L /cow/day, equating to 18% less milk volume (Table 3).

### 3.2. Experiment 2

Number of lactations and DIM for the first and last cows milked were similar within each farm (Table 4). Mean days in milk, parity, and grain intake were similar (*p* > 0.05) between the first and last cows milked. Pasture nutritive value on offer to the first and last cows differed for all farms apart from farm 1; however, the trend in CP and ADF for farm 1 was similar to the other farms with CP being greater and ADF reduced for the first cows as compared to the last cows. Dairy cattle milked first (first 50 cows) in farm 1 had greater (*p* < 0.05) milk yield (8%), protein yield (12%), and solids non-fat yield (4.5%), and 2% less lactose content than those milked last (last 50 cows). For farm 2, dairy cattle milked first had greater (*p* < 0.05) milk yield (11%), SNF yield (7%), and lactose yield (7%), but 2% less protein and 3% less SNF content than cattle milked last. In farm 3, cattle milked first produced milk with 6% greater fat and 3% greater protein content than cattle milked last.

Pasture botanical composition, herbage allowance, pasture intake, and duration of milking for each farm is provided in Table 5. 

**Figure 1:**
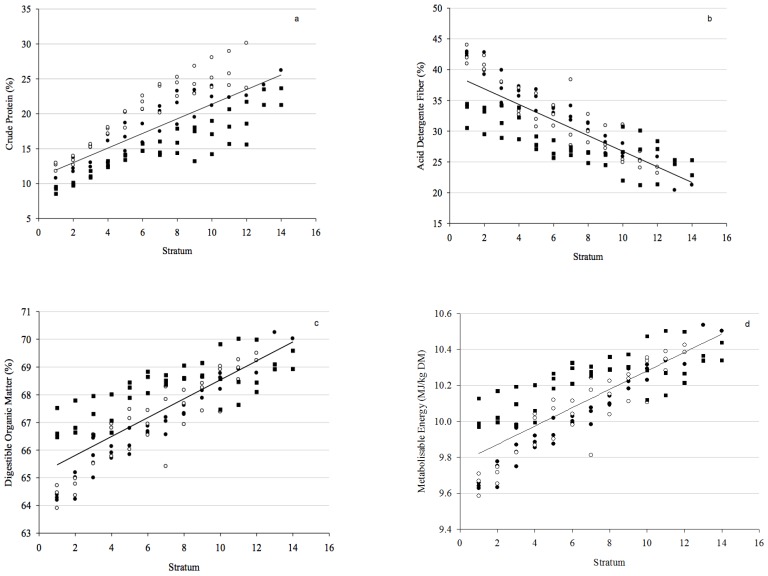
The association between crude protein (CP) (**a**), acid detergent fiber (ADF) (**b**), organic matter digestibility (OMD) (**c**), metabolizable energy (ME) (**d**), and pasture stratum (in cm) for Farm 1 (black circle), Farm 2 (white circle), and Farm 3 (black square). a: 10.9 + 1.05x, R^2^ = 0.58, *p* < 0.0001; b: 39.4 − 1.27x, R^2^ = 0.67, *p* < 0.0001; c: 65.1 + 0.34x, R^2^ = 0.67, *p* < 0.0001; d: 9.8 + 0.05x, R^2^ = 0.67, *p* < 0.0001.

There was a linear increase in CP, OMD, and ME and a decrease in ADF content from the bottom to the top of the pasture sward in all farms (Figure 1). These differences were more pronounced in CP content for the top stratum compared with the bottom stratum (106%).

## 4. Discussion

Our work is the first to together show the association between milking order and milk yield, and milk composition and pasture nutritive value accessed by grazing dairy cattle. The first cows to be milked produced, on average, 18% more milk than those milked last with a range for individual farms of between 14 and 29%. Changes in the nutritive value of pasture that cattle access after milking could be key to these findings. Work from our laboratory [5] with an intermediate herd size (350 cows), showed the crude protein on offer to cattle to decrease by 21% and acid detergent fiber to increase by 15% from the time the first to last cattle accessed pasture voluntarily after 1.7 h of milking duration. In comparison, differences in milk yield between the first and last cows in experiment 2 were less than experiment 1 (10% vs 18%, respectively). This could be explained by the overall changes in pasture nutritive value that occur between seasons, with spring pasture having a greater nutritive value as compared with summer/autumn pasture [4,15] and a potential greater energy deficit in the summer months compared to spring. The greater overall nutritive value of pasture offered to cattle in the spring may have also reduced the differences in milk production from the first to last cattle. Whilst nutritive value and the amount of feed on offer are likely drivers of differences in milk yield with milking order, higher yielding animals may have been more eager to be milked first. However, recently published work [9] isolated the impact of fresh or depleted pasture swards on milk yield and showed a similar reduction in milk yield to those animals milked first or last in the current study. This would suggest that those animals milked first in the current study ingested pasture of greater nutritive value, as per what was on offer, compared with those milked last. Further work is required to elucidate these differences in actual pasture ingested between cattle.

In addition to milk yield, milk component yield tended to be greater for those cattle milked first as compared to the cattle milked last. However, the impact of milking order on milk composition was variable between farms. The greatest differences in milk yield and composition were found in farm 2. Cows that were milked first produced 11% more milk yield, 7% more solids non-fat yield, and 7% more lactose yield than those milked last, and these differences were reflected in changes in pasture height (+45%), CP (+22%), ADF (−19.7%), and OMD (−2.4%) between milking order groups. The main point of contrast for this farm was the more restricted area offered per cow and greater pasture CP% on offer for the first cows. In contrast to other farms, cattle early in the milking order for farm 3 produced 3% greater milk fat and 6% greater milk protein content than those cows milked last, but milk yield was similar between groups. This farm (farm 3) allocated cattle at a lower stocking rate which likely enabled greater selection within the pasture sward by cattle, particularly those later in the milking order, due to a reduced rate of sward depletion. 

There was a large difference in pasture composition across vertical strata of pasture, especially CP and ADF content, between the top and the bottom 2 cm of the sward. In agreement with our findings, Delagarde et al. [4] observed a decrease in CP content (45%) from the top to the bottom 5 cm of the ryegrass sward. Similar trends for other pasture species have also been shown for limpograss, cocksfoot, and orchard grass [16,17,18] alongside herbage digestibility [4,17,18,19,20]. In contrast, NDF, lignin, and non-structural carbohydrate tend to increase from the top to the bottom of the pasture sward [4,15,18,21]. Some studies have reported contrasting findings for pasture composition among strata with poorer growth, or season reducing differences in composition amongst strata [15,18,21], but nitrogen fertilizer application increasing differences in composition between strata [17]. Thus, season and N fertilizer may impact the nutritive value of pasture accessed by cattle as milking progresses, with poorer quality feeds with less leaf having reduced variation in content from top to bottom. To reduce differences in pasture nutritive value consumed across a herd, irrespective of season, it would seem justified that management should ensure that the entire herd should start to graze at the same time. However, greater time off-pasture on standoff facilities can increase plasma cortisol concentrations [22] and risks of hoof injury and lameness [23,24,25], and decrease reproductive efficiency and milk yield [23,26,27,28]. Holding dairy cows on previous pasture allocations or a fraction of that day’s allocation until all cows have arrived post-milking could be a potential strategy to overcome these limitations; however, this strategy would pose its own challenges around overgrazing and/or pasture damage. Individual feeding according to milking order, milk yield, and pasture composition could be used to achieve a better nutrition balance, avoiding underfeeding, reducing nitrogen excretion, and improving milk yield, milk composition, and farm profitability. Each of these strategies requires further investigation related to improving nutrient use efficiency for increased herd productivity. The impact of these strategies on urinary nitrogen excretion also warrant further investigation as the first cows to the paddock accessing high CP forage may contribute the greatest to nitrogen loss from the system.

Daily time on pasture was around 18 to 20.5 h for all the three farms and daytime grazing for last cows was around 32, 22, and 39% lower than first cows in Farms 1, 2, and 3, respectively. The pasture intake of our cows was unlikely to be restricted by time on pasture or nutritive value of pasture as sufficient daytime grazing (8 to 13 h) and low ADF content of pasture (26%) were available, regardless of milking order group. Cows have low grazing activity at night [29], but 8 h of daytime grazing seems to be enough time to ensure similar pasture intake between first and last cows as milk yield is typically reduced when daily time on pasture is less than 8 h [30]. Additionally, the last cows in the current study may have modified their grazing behavior to compensate for reduced time on pasture to achieve similar daily DMI [30,31]. Fiber content of pasture is also relevant to DMI in grazing-based dairy systems as cell walls contribute to rumen fill [32], and it is negatively associated with potential intake [33]. However, pasture intake of first or last cows was unlikely to be impacted by ADF content (24.2 and 27.6%, respectively) as ADF levels were close to the minimal level to provide sufficient fiber to maintain rumen pH, rumen function, cow health, and fat synthesis (21% ADF according to [34]). However, there may be times of the year when milk fat percent is depressed for those cows milked first due to low levels of fiber (low milk fat depression syndrome), particularly in early spring; conversely, low summer pasture quality (high fiber levels) may interact with milking order to limit feed ingested for those cattle. The impact of both scenarios on animal health and production should be the topics of future research.

## 5. Conclusions

There can be an impact of milking order on the nutritive value of pasture offered to dairy cattle together with milk yield and milk composition. Changes in the nutritive value of pasture appear linked with differences in milk yield and milk composition for dairy cattle offered pasture immediately after milking. However, the impact of milking order on milk yield and milk composition was variable between farms and experiments, highlighting the need for further work to capitalize on milking order data. Foremost of these opportunities is to determine the direct link between pasture nutritive value and milk production across dairy herds. Holding dairy cows on previous pasture allocations, or a fraction of that day’s allocation, until all cows have arrived post-milking could be a potential strategy to minimize differences in pasture composition across vertical strata of pasture, especially CP and ADF content. However, this strategy poses its own challenges around animal health and welfare, overgrazing, and/or pasture damage. 

## Figures and Tables

**Table 1:** The number of cows, days in milk (DIM), mean weekly milk yield (L/cow/day), body weight (kg/cow), and grain-based concentrate offered (kg/cow/day) for six farms at the start of experiment 1.

Farm	Number Cows		DIM		Milk Yield (L/cow/day)		Body Weight (kg/cow) ^2^		Grain-Based Concentrate (kg/cow/day) ^2^	
	Mean	SD ^1^	Mean	SD	Mean	SD	Mean	SD	Mean	SD
1	451	40	192	97	27.1	8	555	88	10	3
2	519	50	227	119	21.2	6	-	-	5	1
3	704	33	178	127	24.6	8	-	-	7	2
4	770	41	187	121	26.4	8	-	-	7	2
5	497	5	253	35	13.0	3	-	-	2	1
6	774	36	278	186	15.5	5	475	77	4	3

^1^ Standard deviation. ^2^ 7 days average.

**Table 2:** The number of cows, milk yield (L/cow/day), days in milk (DIM), and grain-based concentrate offered (kg/cow/day) for three focal farms from Tasmania at the start of experiment 2.

Farm	Number of Cows	Milk Yield (L/cow/day)		Days in Milk		Grain-Based Concentrate (kg/cow/day)	
		Mean	SD ^1^	Mean	SD	Mean	SD
1	805	28.7	12.9	128	127	4.3	0.6
2	708	23.7	12.4	311	172	1.9	1.1
3	724	22.9	9.8	137	117	4.3	0.7

^1^ Standard deviation.

**Table 3:** Milk throughput (cows/hour), milking duration (hours/day), and milk yield difference (MYdif) between the first 50 and last 50 cows for each of the 6 farms.

Farm	MYdif (%)	MYdif (L/cow/d)	Number of Cattle		Milking Throughput (cows/h)		Milking Duration (h/d)	
			Mean	SD ^1^	Mean	SD	Mean	SD
1	17	–5.0	476	115	210	38	2.3	0.9
2	19	–3.9	568	172	212	40	2.7	0.9
3	29	–7.5	700	111	231	42	3.2	0.9
4	15	–4.1	776	118	178	29	4.5	0.9
5	14	–2.3	618	82	272	47	2.3	0.6
6	16	–3.4	763	210	232	55	3.4	1.2

^1^ Standard deviation.

**Table 4:** Mean days in milk (d), lactation number, milk yield (L/d) and composition (%), pasture height (cm), and pasture nutritive value accessed for the first 50 and last 50 cows milked for three farms.

Variable	Farm 1				Farm 2				Farm 3			
	First	Last	SE ^1^	*p* Value	First	Last	SE	*p* Value	First	Last	SE	*p* Value
DIM (d)	300	301	16.3	NS	106	107	8.4	NS	218	218	5.2	NS
Number of lactations	1.3	1.3	0.46	NS	2.1	2.3	1.38	NS	3.0	2.5	1.84	NS
MY (L/d)	24.6	22.8	0.54	*	30.4	27.3	0.65	*	23.1	23.2	0.68	NS
Fat (%)	4.2	4.4	0.08	NS	3.7	3.8	0.07	NS	3.7	3.5	0.06	*
Protein (%)	3.7	3.7	0.04	NS	3.8	3.9	0.04	*	3.7	3.6	0.03	*
Somatic Cell Count (×1000)	54.7	70.5	12.42	NS	82.0	100.6	22.88	NS	69.2	70.6	16.48	NS
SNF (%) ^2^	9.4	9.5	0.04	NS	9.5	9.8	0.05	*	9.6	9.5	0.03	NS
Lactose (%)	5.1	5.2	0.02	*	5.0	5.0	0.02	†	5.1	5.1	0.02	NS
Fat yield (kg/d)	1.0	1.0	0.02	NS	1.1	1.0	0.02	†	0.9	0.8	0.03	NS
Protein yield (kg/d)	0.9	0.8	0.02	*	1.1	1.1	0.02	NS	0.8	0.8	0.02	NS
SNF yield (kg/d)	2.3	2.2	0.05	*	2.9	2.7	0.06	*	2.2	2.2	0.06	NS
Lactose yield (kg/d)	1.3	1.2	0.03	†	1.5	1.4	0.03	*	1.2	1.2	8.43	NS
Pasture height (cm)	20.9	18.4	1.6	NS	22.7	15.7	1.6	*	26.9	20.4	1.6	*
Pasture stratum	11.0	10.0	0.8	NS	12.0	8.0	0.8	*	14.0	11.0	0.8	*
Pasture crude protein (%)	23.4	21.9	0.9	NS	28.7	23.5	0.9	*	21.2	18.1	0.9	*
ADF (%) ^3^	25.4	27.6	1.2	NS	23.6	29.4	1.2	*	23.6	25.8	1.2	NS
OMD (%) ^4^	68.9	68.3	0.3	NS	69.4	67.8	0.3	*	69.4	68.8	0.3	NS
ME (MJ/kg/DM) ^5^	10.3	10.2	0.0	NS	10.4	10.2	0.0	*	10.5	10.4	0.0	NS

* *p* < 0.05; †: *p* < 0.10; NS: Not significant (*p >* 0.10); ^1^ Standard Error; ^2^ Solids-not-fat; ^3^ Acid detergent fiber; ^4^ Organic matter digestibility; ^5^ Metabolizable energy.

**Table 5:** Pasture botanical composition (%), herbage allowance, pasture intake, duration milking (h), grain-based concentrate allocated (kg/cow/d), and grain crude protein % for each farm.

Variable	Farm 1	Farm 2	Farm 3	SE^1^	*p* Value
Ryegrass (%)	89.6 ^a^	85.5 ^a^	71.7 ^b^	1.1	*
Clover (%)	5.8 ^b^	10.1 ^ab^	16.1 ^a^	1.1	*
Other species (%)	4.6 ^b^	4.3 ^b^	12.3 ^a^	1.1	*
Area grazed morning paddock (ha)	4.2 ^a^	1.1 ^b^	5.1 ^a^	0.3	*
Pre-grazing height (cm)	20.9 ^b^	22.7 ^ab^	26.9 ^a^	1.4	†
Post-grazing height (cm)	11.5	10.5	12.2	2.0	NS
Pre-grazing mass (kg/DM/ha)	4022 ^b^	4162 ^b^	5641 ^a^	389	*
Post-grazing mass (kg/DM/ha)	2317 ^b^	2729 ^ab^	3688 ^a^	408	*
Herbage allowance (kg DM/cow)	34.1 ^a^	11.9 ^b^	40.1 ^a^	3.8	*
Pasture DMI^2^ (kg/DM/cow)	10.2 ^ab^	4.1 ^b^	13.9 ^a^	1.8	*
Duration milking (h)	2.2 ^b^	1.5 ^c^	3.0 ^a^	0.1	*
Grain (kg/cow/d)	1.9 ^b^	4.2 ^a^	4.3 ^a^	0.03	*
Grain crude protein (%)	5.9	8.4	11.1	-	-

^a‒c^ Means within a row with different superscripts differ (*: *p* < 0.05, †: *p* < 0.10, NS: Not significant and *p* > 0.10); ^1^ Standard Error; ^2^ Dry matter intake (DMI) from morning paddocks.

## References

[1] Doyle P.T., Stockdale C.R., Fuquay J.W., Fox P.F., McSweeney P.L.H. (2011). Dairy farm management systems: Seasonal, pasture-based, dairy cow breeds. Encyclopaedia of Dairy Sciences.

[2] Wade M.H., Carvalho P.F. (2000). Defoliation patterns and herbage intake in grazed pastures. Ecophysiology of Grasslands and the Ecology of Grazing.

[3] Jouven M., Carrère P., Baumont R. (2006). Model predicting dynamics of biomass, structure and digestibility of herbage in managed permanent pastures. 2. Model evaluation. Grass Forage Sci..

[4] Delagarde R., Peyraud J.L., Delaby L., Faverdin P. (2000). Vertical distribution of biomass, chemical composition and pepsin-cellulase digestibility in a perennial ryegrass sward: Interaction with month of year, regrowth age and time of day. Anim. Feed Sci. Technol..

[5] Scott B.A., Clark C.E.F., Camacho A., Golder H., Molfino J., Kerrisk K.L., Lean I., García S.C., Chaves A.V., Hall E. The nutritive value of pasture ingested by dairy cows varies within a herd. Proceedings of the 6th Australasian Dairy Science Symposium.

[6] Barret P.B., Laidlaw A.S., Mayne C.S., Christie H. (2001). Pattern of herbage intake rate and bite dimensions of rotationally grazed dairy cows as sward height declines. Grass Forage Sci..

[7] Botheras N. (2006). The Behaviour and Welfare of Grazing Dairy Cows (Bos Taurus): Effects of Time Away from Pasture and Position in the Milking Order. Ph.D. Thesis.

[8] Beggs D.S., Jongman E.C., Hemsworth P.H., Fisher A.D. (2018). Short communication: Milking order consistency of dairy cows in large Australian herds. J. Dairy Sci..

[9] Clark C.E.F., Kaur R., Millapan L.O., Golder H.M., Thomson P.C., Horadagoda A., Islam R.M., Kerrisk K., Garcia S.C. (2018). The effect of temperate or tropical pasture grazing state and grain-based concentrate allocation on dairy cattle production and behavior. J. Dairy Sci..

[10] Rathore A.K. (1982). Order of cow entry at milking and its relationships with milk yield and consistency of the order. Appl. Anim. Ethol..

[11] Polikarpus A., Kaart T., Mootse H., De Rosa G., Arney D. (2015). Influences of various factors on cows’ entrance order into the milking parlour. Appl. Anim. Behav. Sci..

[12] Grasso F., De Rosa G., Napolitano F., Di Francia A., Bordi A. (2007). Entrance order and side preference of dairy cows in the milking parlour. Ital. J. Anim. Sci..

[13] Berry D.P., McCarthy J. (2012). Genetic and non-genetic factors associated with milking order in lactating dairy cows. Appl. Anim. Behav. Sci..

[14] Ketelaars J.J.M.H., Tolkamp B.J. (1996). Oxygen efficiency and the control of energy flow in animals and humans. J. Anim. Sci..

[15] Brink G.E., Casler M.D., Hall M.B. (2007). Canopy Structure and Neutral Detergent Fiber Differences among Temperate Perennial Grasses. Crop Sci..

[16] Holderbaum J.F., Sollenberger L.E., Quesenberry K.H., Moore J.E., Jones C.S. (1992). Canopy structure and nutritive value of rotationally-grazed limpograss pastures during mid-summer to early autumn. Agron. J..

[17] Duru M. (2003). Effect of nitrogen fertiliser rates and defoliation regimes on the vertical structure and composition (crude protein content and digestibility) of a grass sward. J. Sci. Food Agric..

[18] Griggs T.C., MacAdam J.W., Mayland H.F., Burns J.C. (2007). Temporal and vertical distribution of nonstructural carbohydrate, fiber, protein, and digestibility levels in orchardgrass swards. Agron. J..

[19] Burns J.C., Pond K.R., Fisher D.S. (1991). Effects of grass species on grazing steers: II. Dry matter intake and digesta kinetics. J. Anim. Sci..

[20] Fisher D.S., Burns J.C., Pond K.R., Mochrie R.D., Timothy D.H. (1991). Effects of grass species on grazing steers: 1. Diet composition and ingestive mastication. J. Anim. Sci..

[21] Nave R.L.G., Sulc R.M., Barker D.J., St-Pierre N. (2014). Changes in Forage Nutritive Value among Vertical Strata of a Cool-Season Grass Canopy. Crop Sci..

[22] Fisher A.D., Verkerk G.A., Morrow C.J., Matthew L.R. (2002). The effects of feed restriction and lying deprivation on pituitary-adrenal axis regulation in lactating cows. Livest. Prod. Sci..

[23] Coulon J.B., Pradel P., Cochard T., Poutrel B. (1998). Effect of extreme walking conditions for dairy cows on milk yield, chemical composition, and somatic cell count. J. Dairy Sci..

[24] Chaplin S.J., Ternent H.E., Offer J.E., Logue D.N., Knight C.H. (2000). A comparison of hoof lesions and behaviour in pregnant and early lactation heifers at housing. Vet. J..

[25] Galindo F., Broom D.M. (2000). The relationships between social behaviour of dairy cows and the occurrence of lameness in three herds. Res. Vet. Sci..

[26] Hassall S.A., Ward W.R., Murray R.D. (1993). Effects of lameness on the behaviour of cows during the summer. Vet. Rec..

[27] Barkema H.W., Westrik J.D., Van Keulen K.A.S., Schukken Y.H., Brand A. (1994). The effects of lameness on reproductive performance, milk production and culling in Dutch dairy farms. Prev. Vet. Med..

[28] Melendez P., Bartolome J., Archbald L.F., Donovan A. (2003). The association between lameness, ovarian cysts and fertility in lactating dairy cows. Theriogenology.

[29] Pérez-Ramírez E., Peyraud J.L., Delagarde R. (2009). Restricting daily time at pasture at low and high pasture allowance: Effects on pasture intake and behavioral adaptation of lactating dairy cows. J. Dairy Sci..

[30] Clark C.E.F., McLeod K.L.M., Glassey C.B., Gregorini P., Costall D.A., Betteridge K., Jago J.G. (2010). Capturing urine while maintaining pasture intake, milk production, and animal welfare of dairy cows in early and late lactation. J. Dairy Sci..

[31] Kennedy E., McEvoy M., Murphy J.P., O’Donovan M. (2009). Effect of restricted access time to pasture on dairy cow milk production, grazing behavior, and dry matter intake. J. Dairy Sci..

[32] Jung H.G., Allen M.S. (1995). Characteristics of plant cell walls affecting intake and digestibility of forages by ruminants. J. Anim. Sci..

[33] Vazquez O.P., Smith T.R. (2000). Factors affecting pasture intake and total dry matter intake in grazing dairy cows. J. Dairy Sci..

[34] NRC (1989). Nutrient Requirements of Dairy Cattle.

